# Potential Rhodopsin- and Bacteriochlorophyll-Based Dual Phototrophy in a High Arctic Glacier

**DOI:** 10.1128/mBio.02641-20

**Published:** 2020-11-24

**Authors:** Yonghui Zeng, Xihan Chen, Anne Mette Madsen, Athanasios Zervas, Tue Kjærgaard Nielsen, Adrian-Stefan Andrei, Lars Chresten Lund-Hansen, Yongqin Liu, Lars Hestbjerg Hansen

**Affiliations:** a Department of Environmental Science, Aarhus University, Roskilde, Denmark; b Aarhus Institute of Advanced Studies, Aarhus, Denmark; c Department of Engineering, Aarhus University, Aarhus, Denmark; d The National Research Centre for the Working Environment, Copenhagen, Denmark; e Department of Plant and Environmental Sciences, University of Copenhagen, Frederiksberg, Denmark; f Limnological Station, Institute of Plant and Microbial Biology, University of Zurich, Kilchberg, Switzerland; g Arctic Research Center, Department of Bioscience, Aarhus University, Aarhus, Denmark; h Key Laboratory of Tibetan Environment Changes and Land Surface Processes, Institute of Tibetan Plateau Research, Chinese Academy of Sciences, Beijing, China; i University of Chinese Academy of Sciences, Beijing, China; University of Georgia

**Keywords:** phototrophy, glacial bacteria, bacteriochlorophyll, rhodopsin, genome evolution

## Abstract

Conserving additional energy from sunlight through bacteriochlorophyll (BChl)-based reaction center or proton-pumping rhodopsin is a highly successful life strategy in environmental bacteria. BChl and rhodopsin-based systems display contrasting characteristics in the size of coding operon, cost of biosynthesis, ease of expression control, and efficiency of energy production. This raises an intriguing question of whether a single bacterium has evolved the ability to perform these two types of phototrophy complementarily according to energy needs and environmental conditions. Here, we report four *Tardiphaga* sp. strains (*Alphaproteobacteria*) of monophyletic origin isolated from a high Arctic glacier in northeast Greenland (81.566° N, 16.363° W) that are at different evolutionary stages concerning phototrophy. Their >99.8% identical genomes contain footprints of horizontal operon transfer (HOT) of the complete gene clusters encoding BChl- and xanthorhodopsin (XR)-based dual phototrophy. Two strains possess only a complete XR operon, while the other two strains have both a photosynthesis gene cluster and an XR operon in their genomes. All XR operons are heavily surrounded by mobile genetic elements and are located close to a tRNA gene, strongly signaling that a HOT event of the XR operon has occurred recently. Mining public genome databases and our high Arctic glacial and soil metagenomes revealed that phylogenetically diverse bacteria have the metabolic potential of performing BChl- and rhodopsin-based dual phototrophy. Our data provide new insights on how bacteria cope with the harsh and energy-deficient environment in surface glacier, possibly by maximizing the capability of exploiting solar energy.

## Abstract (precis)

Over the course of evolution for billions of years, bacteria that are capable of light-driven energy production have occupied every corner of surface Earth where sunlight can reach. Only two general biological systems have evolved in bacteria to be capable of net energy conservation via light harvesting: one is based on the pigment of (bacterio-)chlorophyll and the other is based on proton-pumping rhodopsin. There is emerging genomic evidence that these two rather different systems can coexist in a single bacterium to take advantage of their contrasting characteristics in the number of genes involved, biosynthesis cost, ease of expression control, and efficiency of energy production and thus enhance the capability of exploiting solar energy. Our data provide the first clear-cut evidence that such dual phototrophy potentially exists in glacial bacteria. Further public genome mining suggests this understudied dual phototrophic mechanism is possibly more common than our data alone suggested.

## OBSERVATION

Over billions of years of evolution, phototrophic bacteria capable of light-driven energy generation have occupied every corner of surface Earth where solar irradiation can reach. Only two general biological systems are known in bacteria to be capable of net energy conservation from light harvesting: one is based on bacteriochlorophyll (BChl; chlorophyll in the case of *Cyanobacteria*) and the other is based on proton-pumping rhodopsin (1). BChl-based light harvesting relies on a complex system consisting of dozens of proteins and pigments to form reaction center and antenna complex. In contrast, the rhodopsin-based system requires only a few genes to operate, including a key pair of genes, the rhodopsin gene and the carotenoid oxygenase gene *blh/brp* for retinal biosynthesis (2), albeit at a much lower efficiency in energy production than the BChl-based system (3).

BChl- and rhodopsin-based systems display contrasting characteristics in the size of coding operon, cost of biosynthesis, ease of expression control, and efficiency of energy production. This raises an intriguing question of whether a single bacterium can employ both types of phototrophy to take advantage of their complementary properties in order to increase the flexibility in energy production. Given the high abundance and frequent coexistence of BChl-based phototrophs and rhodopsin-based phototrophs in the same environment, for instance, oceans (4), and given that phototrophy-related genes frequently occurred in extrachromosomal genetic elements, like photosynthesis gene cluster on plasmids (5, 6) or chromid (7) and proteorhodopsin gene in viral genomes (8), BChl- and rhodopsin-based dual phototrophic bacteria very likely have evolved in nature, awaiting discovery.

Indeed, there is emerging genomic evidence for such dual phototrophy. Recently, three *Roseiflexus* genomes (*Chloroflexi* phylum) from spring microbial mats were found to contain both *pufM* (encoding the M subunit of reaction center) and xanthorhodopsin (XR)-like genes (9), including two metagenome-assembled genomes (MAGs) of *Roseiflexus* spp. OTU-1 and OTU-6 (10) and one from the isolate *Roseiflexus* sp. RS-1 (11), albeit it is unclear whether their rhodopsins function as a bona fide proton pump, owing to the absence of the key carotenoid oxygenase gene *blp/brh* that is often located in the genomic neighborhood of the rhodopsin gene (10).

### Discovery of glacial bacteria with potential dual phototrophy.

**FIG 1:**
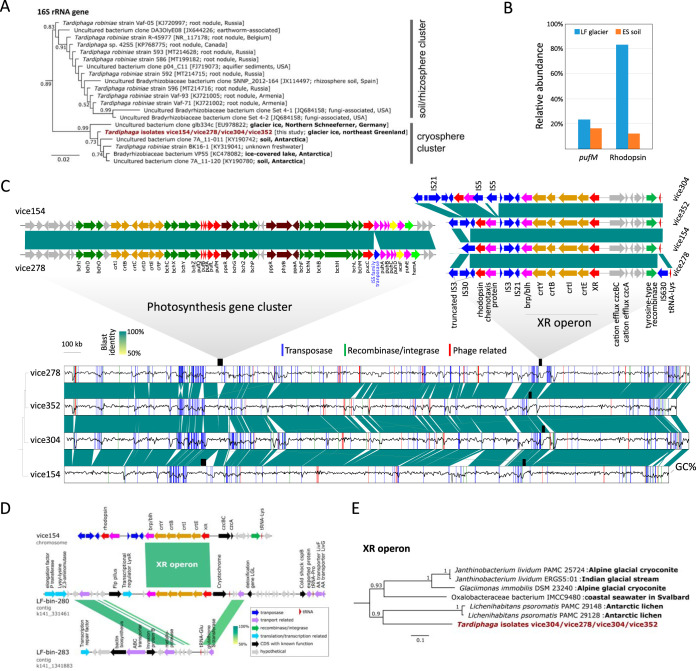
(A) Maximum-likelihood phylogeny of 16S rRNA genes. (B) Abundance of *pufM* and rhodopsin genes in the “Little Firn” glacier (LF) and nearby exposed soil (ES) metagenomes, calculated as the number of mapped reads normalized to per-kb gene length divided by the normalized number of reads mapped to the housekeeping gene *recA*. (C) Genome synteny and sequence similarities of *Tardiphaga* sp. strains vice154, vice278, vice304, and vice352. The GenBank accession number and source environment of each 16S rRNA gene sequence are shown in parentheses on the tree. The architecture of photosynthesis gene cluster and xanthorhodopsin (XR) operon are highlighted. Gaps in the alignment show nonconserved regions caused by mobilome-related activities, including transposase, recombinase, integrase, or phage-related genes, the locations of which are highlighted in the genome. All genomes are complete and start at the replication origin locus. The black wavy line inside each genome (shown as boxes) represents GC content calculated in a window of 1 kb. The relationship between strains was estimated as a distance-based genome tree in the bottom left using MASH (https://github.com/marbl/Mash). Color bar, BLAST identities. (D) Genome synteny between two contigs from the XR-bearing glacial *Tardiphaga* MAG LF-bin-280 and the nonphototrophic glacial *Tardiphaga* MAG LF-bin-283 in reference to the genome of strain vice154. MAG, metagenome-assembled genome. (E) Maximum-likelihood phylogeny of the whole XR operon of *Tardiphaga* isolates and their top tBLASTn hits in NCBI’s genome database; see Fig. S5 for the full version of the tree.

Aiming to provide further pure culture and direct evolutionary evidence for dual phototrophy, we conducted both cultivation and metagenomics survey on the microbial communities in the “Lille Firn” glacier (LF) and nearby exposed soil (ES) in northeast Greenland (81.566° N, 16.363° W; Fig. S1). A collection of isolates of aerobic phototrophic bacteria was created from the LF surface glacial ice sample. Four pinkish colonies (designated strains vice154, vice278, vice304, and vice352) were further examined due to high similarities in their profiles on the matrix-assisted laser desorption/ionization–time of flight mass spectrometer and due to the observation that vice154 and vice278 displayed weak BChl fluorescence signals inside the colony infrared imaging system (12). The 16S rRNA genes of these four strains are 100% identical and share 96.5% identity to Tardiphaga robiniae LMG 26467^T^ of the genus *Tardiphaga* of *Alphaproteobacteria* (13), indicating that they represent a novel species in the cryospheric cluster of *Tardiphaga* (Fig. 1A). In the glacial bacterial community (LF), members of *Tardiphaga* accounted for 0.017% (17/101,183 reads) (Fig. S2). No *Tardiphaga*-affiliated read was found in ES (*n* = 10,060). Thus, *Tardiphaga* represents one of the least abundant groups and occurs only in LF.

10.1128/mBio.02641-20.2 FIG S1 Photographs showing the sampling site, fieldwork, and visual inspection of a sampled ice block. Download FIG S1, PDF file, 1.6 MB. Copyright © 2020 Zeng et al. 2020 Zeng et al. This content is distributed under the terms of the Creative Commons Attribution 4.0 International license.

10.1128/mBio.02641-20.3 FIG S2 Composition of total bacterial communities in LF and ES at the phylum level (A) highlighting *Alphaproteobacteria* that contain the *Tardiphaga* genus (B). Download FIG S2, PDF file, 0.3 MB. Copyright © 2020 Zeng et al. 2020 Zeng et al. This content is distributed under the terms of the Creative Commons Attribution 4.0 International license.

Despite their monophyletic origin as reflected by the genome pairwise average nucleotide identity of >99.8% and the highly conserved genome synteny (Fig. 1C), these four strains differed in both genome size and GC content (Table S1). These differences were primarily caused by insertions and deletions (Fig. 1C), including a 45.7-kb photosynthesis gene cluster that is present in vice154 and vice278 but absent in vice304 and vice352. These two photosynthesis gene clusters differ only in the insertion of an IS*5* family transposase gene between *pucC* and *puhA* in vice278 (Fig. 1C). No mobilome-related genes were found in the proximity of the photosynthesis gene cluster in both vice154 and vice278 (Fig. S3), suggesting that photosynthesis gene cluster is an ancient trait in their ancestor that later was lost in vice304 and vice352.

10.1128/mBio.02641-20.4 FIG S3 Annotated genomic regions surrounding the photosynthesis gene cluster in *Tardiphaga* strains vice154 and vice278 in comparison to strains vice304 and vice352 that do not contain a photosynthesis gene cluster. Download FIG S3, PDF file, 1.5 MB. Copyright © 2020 Zeng et al. 2020 Zeng et al. This content is distributed under the terms of the Creative Commons Attribution 4.0 International license.

10.1128/mBio.02641-20.7 TABLE S1 Summary of complete genomes of the four *Tardiphaga* strains isolated from the “Little Firn” glacier in northeast Greenland. Download Table S1, PDF file, 0.2 MB. Copyright © 2020 Zeng et al. 2020 Zeng et al. This content is distributed under the terms of the Creative Commons Attribution 4.0 International license.

All four genomes contain an XR operon encoding XR-based phototrophy with the same gene arrangement (*XR-crtEIBY-brp*) and almost 100% identical nucleotide sequences (only one base difference out of 6,257 sites occurring in vice304) (Fig. 1C). The predicted XR protein sequence contains most of the conserved sites including key residues as proton acceptor and donor (Fig. S4), suggesting that it very likely encodes a functional proton pump. Interestingly, there are an additional unclassified rhodopsin gene and a putative methyl-accepting chemotaxis gene located immediately upstream of the XR operon and flanked by insertion (IS) elements at both sides.

10.1128/mBio.02641-20.5 FIG S4 Protein sequence alignment showing the presence of residues in *Tardiphaga*’s xanthorhodopsin that are key to its function as a proton pump. Download FIG S4, PDF file, 2.4 MB. Copyright © 2020 Zeng et al. 2020 Zeng et al. This content is distributed under the terms of the Creative Commons Attribution 4.0 International license.

10.1128/mBio.02641-20.6 FIG S5 Maximum-likelihood phylogeny of the whole XR operon of *Tardiphaga* isolates and their top tBLASTn hits in NCBI’s genome database. Download FIG S5, PDF file, 0.2 MB. Copyright © 2020 Zeng et al. 2020 Zeng et al. This content is distributed under the terms of the Creative Commons Attribution 4.0 International license.

The XR operon is located near the tRNA-Lys gene in all four genomes. Bacterial tRNA genes are considered recombination hot spot region in bacterial genomes (14). Thus, the presence of multiple IS elements and a tRNA gene in the vicinity of the XR operon strongly indicates that a horizontal operon transfer (HOT) event of the XR operon has occurred in these four strains. This was further supported by reconstruction of two *Tardiphaga* MAGs (LF-bin-280 with an XR operon and LF-bin-283 without an XR operon; Table 1), where HOT of a complete XR operon was recorded at a highly homologous region next to the tRNA-Glu gene (Fig. 1D). Since the first discovery of proteorhodopsins in the oceans (15), there has been a growing body of phylogeny-based evidence for horizontal transfer of rhodopsin genes occurring between prokaryotes (16–18). Our data provide further clear-cut evidence for HOT of the rhodopsin gene operon occurring in a natural microbial community.

**TABLE 1:** Summary of metagenome-assembled genomes (MAGs) reconstructed from the metagenomes of the “Little Firn” glacier (LF) and nearby exposed soil (ES) that contain genes related to bacteriochlorophyll- or rhodopsin-based phototrophy^*a*^

MAG	Lineage		Size (Mbp)	No. of contigs	Completeness (%)	Contamination (%)	Phototrophy					
	(Sub)phylum	Genus					*pufM*	PR	ActR	BR	XR	HeR
LF-bin-79	*Alphaproteobacteria*	*Rubritepida*	3.78	585	95.51	3.73	●				●	
LF-bin-280	*Alphaproteobacteria*	*Tardiphaga*	5.55	642	91.96	7.88	●				●	
LF-bin-283	*Alphaproteobacteria*	*Tardiphaga*	4.50	785	92.36	8.02	●					
LF-bin-46	*Betaproteobacteria*	*Ramlibacter*	4.05	336	95.48	4.74	●		●			
LF-bin-10	*Gammaproteobacteria*	n.d.	2.55	141	92.16	2.56		●				
LF-bin-172	*Gammaproteobacteria*	n.d.	3.55	274	91.13	0.99					●	
LF-bin-25	*Actinobacteria*	*Aeromicrobium*	4.22	400	94.99	5.85					●	
LF-bin-295	*Actinobacteria*	*Aeromicrobium*	5.01	519	100	7.52					●	●
LF-bin-300	*Actinobacteria*	n.d.	3.77	278	95.97	2.05					●	●
LF-bin-354	*Actinobacteria*	n.d.	2.42	100	96.17	3.64					●	
LF-bin-355	*Bacteroidetes*	*Ferruginibacter*	3.68	394	91.9	1.07				●		
LF-bin-258	*Bacteroidetes*	*Flavobacterium*	2.95	172	94.92	5.24				●		
LF-bin-351	*Bacteroidetes*	*Flavobacterium*	3.28	269	94.16	3.86				●		
LF-bin-500	*Bacteroidetes*	n.d.	2.94	211	95.95	0.95				●		
LF-bin-319	*Bacteroidetes*	*Pedobacter*	3.00	296	90.03	3.99				●		
LF-bin-240	*Bacteroidetes*	*Pseudopedobacter*	3.34	191	91.39	6.54				●		
LF-bin-127	*Oligoflexia*	*Bdellovibrio*	2.86	186	91.39	0.9		●				
LF-bin-439	*Oligoflexia*	*Bdellovibrio*	3.27	192	95.21	4.68					●	
LF-bin-57	*Oligoflexia*	*Bdellovibrio*	2.76	42	90.32	0.9					●	
LF-bin-124	*Oligoflexia*	n.d.	2.33	259	90.69	4.95					●	
LF-bin-178	*Oligoflexia*	n.d.	4.04	82	91.89	1.79					●	
LF-bin-339	*Gemmatimonadetes*	n.d.	3.78	254	90.61	3.3	●				●	
ES-bin-98	*Alphaproteobacteria*	n.d.	3.50	252	95.22	2.55	●				●	
ES-bin-147	*Armatimonadetes*	n.d.	4.22	495	92.2	2.04					●	
ES-bin-166	*Bacteroidetes*	n.d.	4.04	68	94.46	0.71					●	
ES-bin-26	*Bacteroidetes*	n.d.	5.43	717	90.76	1.36				●		
ES-bin-22	*Cyanobacteria*	*Leptolyngbya*	5.12	603	91.39	3.97					●	
ES-bin-313	*Cyanobacteria*	n.d.	4.25	198	95.73	4.56				●		

a Only MAGs that are >90% complete with <10% contamination are shown. See Table S2 for the full list of MAGs (>50% complete and <10% contamination). PR, proteorhodopsin; XR, xanthorhodopsin; ActR, actinorhodopsin; BR, bacteriorhodopsin; HeR, heliorhodopsin (a recently discovered new type of rhodopsin [26]); n.d., not determined. The classification of rhodopsin genes was based on phylogenetic analysis using a comprehensive collection of rhodopsin genes as the reference data set (see Text S1).

10.1128/mBio.02641-20.1 TEXT S1 Detailed materials and methods with a note on the negative results from cultivation of *Tardiphaga* stains in liquid media and colony pigment analysis. Download Text S1, DOCX file, 0.03 MB. Copyright © 2020 Zeng et al. 2020 Zeng et al. This content is distributed under the terms of the Creative Commons Attribution 4.0 International license.

10.1128/mBio.02641-20.8 TABLE S2 Summary of metagenome-assembled genomes (MAGs) from the LF and ES metagenomes. Download Table S2, XLSX file, 0.05 MB. Copyright © 2020 Zeng et al. 2020 Zeng et al. This content is distributed under the terms of the Creative Commons Attribution 4.0 International license.

**FIG 2:**
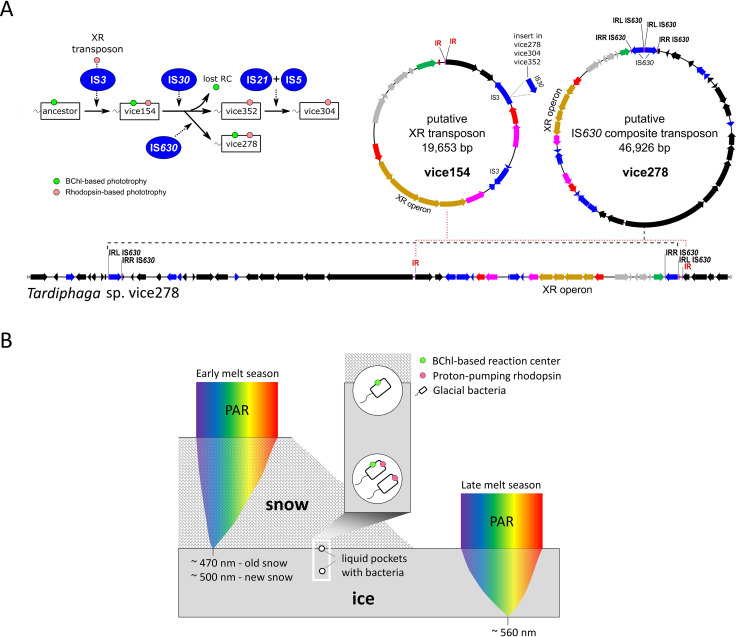
(A) Hypothetical evolutionary path of the *Tardiphaga* isolates from their common ancestor based on the IS insertion patterns (left) and two transposons proposed to drive the movement of the xanthorhodopsin (XR) operon (right). The genomic region surrounding the XR operon in *Tardiphaga* strain vice278 was shown to highlight the distribution of direct or inverted repeats that are required to form the putative transposons. RC, reaction center; IR, inverted repeat; DR, direct repeat, IRL, inverted repeat-left; IRR, inverted repeat-right. (B) A model for light availability in snow and ice and hypothesized niche partitioning of BChl- and rhodopsin-based dual phototrophy versus only BChl- or rhodopsin-based single phototrophy. The snowpack and icepack are depicted to be of such an ideal thickness that the light spectrum with the lowest extinction coefficient reaches the exact bottom of snowpack or icepack. PAR, photosynthetically active radiation. Note that new and old snows have different spectral distribution for PAR.

In our *Tardiphaga* strains, HOT of the XR operon was likely driven by transposon activities as indicated by the presence of an integrase gene (tyrosine-type recombinase family) and direct or inverted repeats in adjacent genomic regions of the integrase gene. These repeats can serve as attachment sites of the recombinase located on the predicted XR transposon and IS*630* composite transposon (Fig. 2A). The putative XR transposon is conserved and thus may occur in all *Tardiphaga* strains. Strain vice278 possesses an additional IS*630* family transposase between the XR operon and the tRNA-Lys gene, an identical copy of which is located 45.7 kb apart upstream of the XR operon (Fig. 2A). These two identical IS*630* genes can serve as inverted repeats for the formation of the putative IS*630* composite transposon. Given that the nucleotide sequences of the whole XR operon in these four *Tardiphaga* genomes are almost 100% identical, the acquisition of an XR operon in their ancestor certainly occurred before the divergence of photosynthesis gene cluster.

### Ecological importance and wide distribution of potential dual phototrophs.

Glaciers and ice sheets cover 10% of the land surface of the Earth, hosting an enormous diversity of microbes (19), among which light-driven metabolisms in BChl-based anoxygenic phototrophs have been proposed to have the potential of significantly influencing glacial carbon flux (20). BChl- and rhodopsin-based dual phototrophy can theoretically further reduce the consumption of organic matter for energy production in glacial bacteria by increasing the flexibility and efficiency in conserving light energy. Such dual phototrophy, if proved to function *in situ* in glacial microbial communities, could amplify the ecological importance of anoxygenic phototrophs in the glacial ecosystem.

We assessed the abundance of BChl-based phototrophs and rhodopsin-based phototrophs by targeting *pufM* and rhodopsin genes in the metagenomes of ES and LF. BChl-based phototrophs in both samples were comparable with LF showing a slightly higher abundance (23.3%) (Fig. 1B). However, rhodopsin-based phototrophs were almost 7-fold more abundant in LF than in ES (83.3% versus 12.2%, Fig. 1B), suggesting that rhodopsin-based phototrophs may play a more important role in supraglacial environments than BChl-based phototrophs, in line with the fact that rhodopsin-based phototrophy requires less energy and fewer components for assembly and thus probably responds faster to environmental changes than BChl-based phototrophy.

Dual phototrophy can be particularly advantageous in an extreme environment like the high Arctic glacial surface where phototrophic bacteria may have to exploit all available solar radiation for energy production. Given the advantage of dual phototrophy over single phototrophy in light harvesting, it is unclear why photosynthesis gene cluster was selectively lost in *Tardiphaga* sp. strains vice304 and vice352. We proposed two theories to explain this phenomenon, i.e., niche differentiation and gene mutation.

XR has maximum absorption in green light (9), while BChl and accessory carotenoids in anoxygenic phototrophs mostly absorb blue and infrared light (21). Different light wavelengths are attenuated differently through snow and ice. Under an ideal condition without impurities, gaps, and spatial heterogeneities occurring in snow and ice, blue light tends to reach the deepest into snow (22, 23), while green light at approximately 560 nm has the lowest attenuation coefficient within ice (24). Thus, different niches exist in glacial surface in terms of light intensity and quality, which may select for phototrophic bacteria with different preferences for light spectra (Fig. 2B). The loss of BChl-based phototrophy may also occur through random mutations in key photosynthetic genes, caused by, for instance, the activities of transposases, as we observed inside the photosynthesis gene cluster of strain vice278 (Fig. 1C).

There are nine MAGs reconstructed in this study that contain both *pufM* and rhodopsin genes (Table 1 and Table S2), including five from *Alphaproteobacteria*, three from *Gammaproteobacteria*, and one from *Gemmatimonadetes*, among which all but one were recovered from the LF glacier sample. To test if dual phototrophy is exclusively occurring in supraglacial environments, we further searched public databases (NCBI and ENA, *n* = 215,874; see Text S1) for bacterial genomes of similar dual phototrophy potential (BChl-based reaction center and proton-pumping rhodopsin). We found 3,442 XR/proteorhodopsin-like and 1,521 *pufM*-like tBLASTn hits (Table S3). Fifty-five genomes were predicted to contain both *pufM* and rhodopsin genes (Table S3) with the majority (*n* = 40) belonging to *Alphaproteobacteria*. Interestingly, there are also four *Chloroflexi*, three *Bacteroidetes*, two Deltaproteobacteria, two *Gammaproteobacteria*, and one *Actinobacteria* genome. Given the quality concern on the incomplete genomes deposited into public databases (25), it is unclear whether there is any composite among these genomes, especially those from *Bacteroidetes* and *Actinobacteria*, where BChl-based phototrophy has not yet been reported. The isolation sources of these genomes cover various environments (Table S3), including freshwater, seawater, groundwater, hot spring, microbial mat/biofilm, soil, sediment, plant surface, and cryosphere (alpine/polar; *n* = 24), indicating that dual phototrophy is likely present in a wide range of bacteria and in a large variety of natural environments beyond glaciers.

10.1128/mBio.02641-20.9 TABLE S3 Survey of *pufM* and rhodopsin genes in the genomes deposited into NCBI’s Microbial Genome and ENA’s WGS databases and the list of public genomes containing both *pufM*-like and rhodopsin-like genes. Download Table S3, XLSX file, 0.8 MB. Copyright © 2020 Zeng et al. 2020 Zeng et al. This content is distributed under the terms of the Creative Commons Attribution 4.0 International license.

Dual phototrophy is clearly not a metabolic trait that evolved only in glacial bacteria, albeit the four glacial *Tardiphaga* strains in this study and their XR operons both show cryospheric origins (Fig. 1E). We investigated whether these *Tardiphaga* strains have other metabolic traits that may enable them to adapt to the high Arctic glacial environment. Strikingly, all four genomes contain RuBisCO genes, a phosphoribulokinase gene, and a soluble methane monooxygenase gene (Table S4), pointing to the metabolic potentials of photoautotrophy and methanotrophy. We have so far failed to grow these *Tardiphaga* strains in liquid media and observed the expression of neither BChl nor XR under the tested laboratory growth conditions (Text S1). Further growth optimization and physiological data are warranted to verify their dual phototrophy and other metabolic potentials and to understand how these two types of phototrophy coordinate in their metabolic networks.

10.1128/mBio.02641-20.10 TABLE S4 Survey of key functional genes with biogeochemical or energetic importance in the genomes of *Tardiphaga* isolates. The gene list was compiled from the FunGen pipeline (http://fungene.cme.msu.edu/) and the authors’ own collection. Download Table S4, PDF file, 0.4 MB. Copyright © 2020 Zeng et al. 2020 Zeng et al. This content is distributed under the terms of the Creative Commons Attribution 4.0 International license.

Materials and methods are available as supplemental material in Text S1.

### Sequence data availability.

Genomes, metagenomes, and raw reads were deposited into GenBank under BioProject numbers PRJNA548505 and PRJNA552582.
