# Supplementary material for: Potential Rhodopsin- and Bacteriochlorophyll-Based Dual Phototrophy in a High Arctic Glacier
Source: mBio. 2020 Nov 24;11(6):e02641-20. doi: 10.1128/mBio.02641-20 (PMC7701988; doi:10.1128/mBio.02641-20)
Supplement: FIG S2 [file mBio.02641-20-sf002.pdf]

A

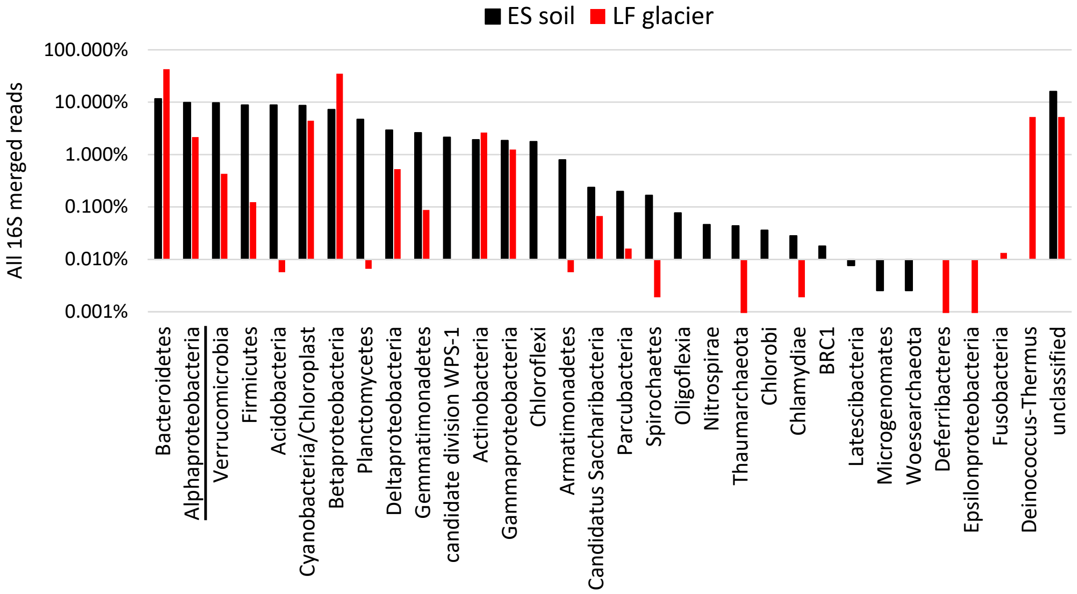

B

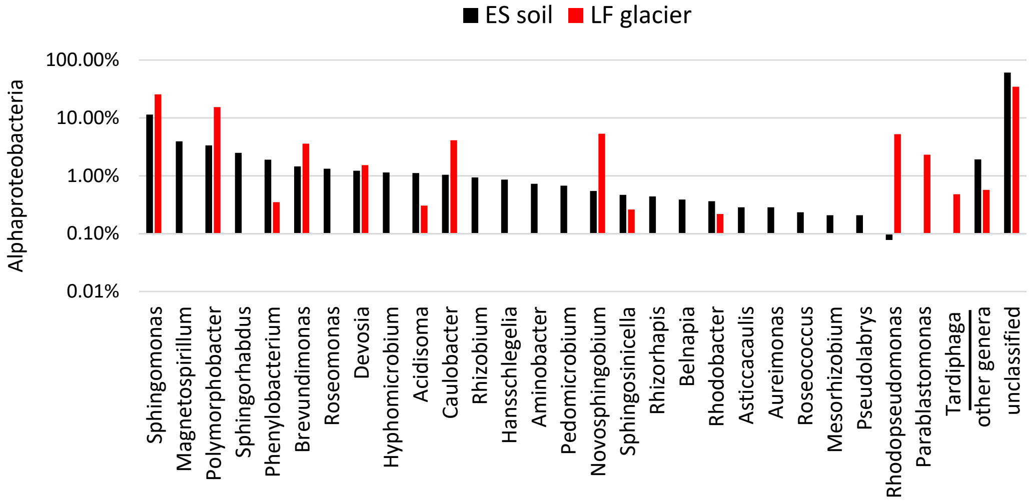

Figure S2 Composition of total bacterial communities in LF and ES at the phylum level (A) highlighting Alphaproteobacteria that contains the *Tardiphaga* genus (B).
