# Supplementary material for: Potential Rhodopsin- and Bacteriochlorophyll-Based Dual Phototrophy in a High Arctic Glacier
Source: mBio. 2020 Nov 24;11(6):e02641-20. doi: 10.1128/mBio.02641-20 (PMC7701988; doi:10.1128/mBio.02641-20)
Supplement: TABLE S1 [file mBio.02641-20-st001.pdf]

Table S1 Summary of the complete genomes of the four *Tardiphaga* strains isolated from the "Little Firn" glacier in northeast Greenland. PGC, photosynthesis gene cluster; XR, xanthorhodopsin. CDS, coding sequence.

Isolate	Genome Size (bp)	GC content	Number of genes						PGC	XR	GenBank accession no.
			rRNA operon	tRNA	Protein CDSs	Trans-posase	Phage-related	Recombinase /integrase			
vice154	5,609,510	63.59%	2	51	5,001	109	18	16	+	+	CP041399
vice278	5,806,756	63.36%	2	53	5,196	222	38	28	+	+	CP041400
vice304	5,788,809	63.28%	2	53	5,192	207	28	33	-	+	CP041402
vice352	5,678,525	63.37%	2	52	5,094	180	41	26	-	+	CP041401
