# Supplementary material for: Potential Rhodopsin- and Bacteriochlorophyll-Based Dual Phototrophy in a High Arctic Glacier
Source: mBio. 2020 Nov 24;11(6):e02641-20. doi: 10.1128/mBio.02641-20 (PMC7701988; doi:10.1128/mBio.02641-20)
Supplement: FIG S4 [file mBio.02641-20-sf004.pdf]

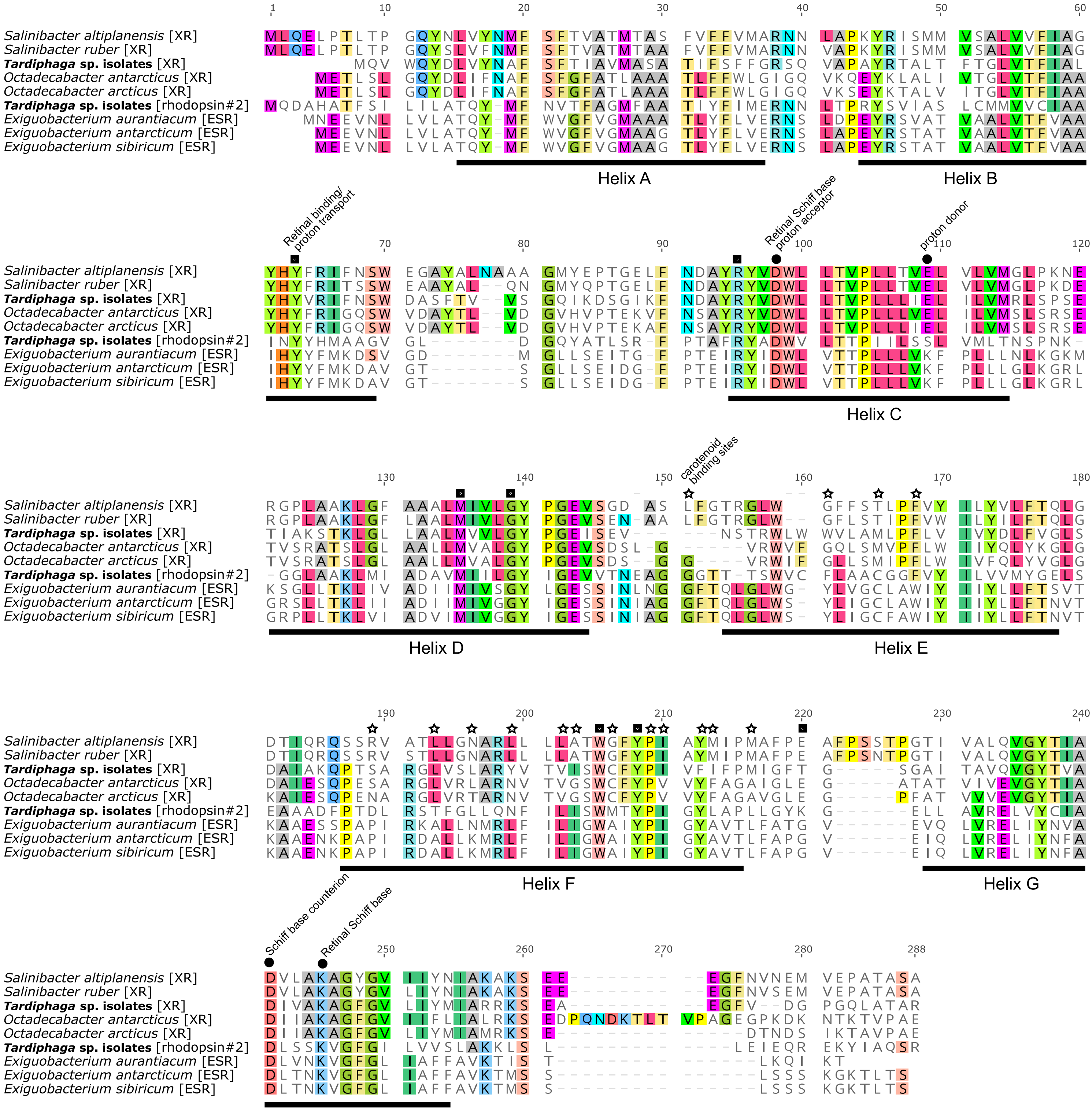

Figure S4 Protein sequence alignment showing the presence of residues in *Tardiphaga*'s xanthorhodopsin that are key to its function as a proton pump.
