# Supplementary material for: Potential Rhodopsin- and Bacteriochlorophyll-Based Dual Phototrophy in a High Arctic Glacier
Source: mBio. 2020 Nov 24;11(6):e02641-20. doi: 10.1128/mBio.02641-20 (PMC7701988; doi:10.1128/mBio.02641-20)
Supplement: TEXT S1 [file mBio.02641-20-s0001.docx]

**Potential Rhodopsin and Bacteriochlorophyll-Based Dual Phototrophy in a High Arctic Glacier**

Yonghui Zeng^1,2 * §^, Xihan Chen^3^, Anne Mette Madsen^4^, Athanasios Zervas^1^, Tue Kjærgaard Nielsen^5^, Adrian-Stefan Andrei^6^, Lars Chresten Lund-Hansen^7^, Yongqin Liu^8,9^, Lars Hestbjerg Hansen^1,5^

^1^Department of Environmental Science, Aarhus University, Roskilde 4000, Denmark; ^2^Aarhus Institute of Advanced Studies, Aarhus 8000, Denmark; ^3^Department of Engineering, Aarhus University, Aarhus 8000, Denmark; ^4^The National Research Centre for the Working Environment, Copenhagen 2100, Denmark; ^5^Department of Plant and Environmental Science, University of Copenhagen, Frederiksberg 1871, Denmark; ^6^Limnological Station, Institute of Plant and Microbial Biology, University of Zurich, Kilchberg 8802, Switzerland; ^7^Arctic Research Center, Department of Bioscience, Aarhus University, Aarhus 8000, Denmark; ^8^Institute of Tibetan Plateau Research, Chinese Academy of Sciences, Beijing 100101, China; ^9^University of Chinese Academy of Sciences, Beijing 100049, China; ^§^Present address: Department of Plant and Environmental Science, University of Copenhagen, Frederiksberg 1871, Denmark.

***Correspondence**:

Yonghui Zeng, Department of Plant and Environmental Science, University of Copenhagen, Frederiksberg 1871, Denmark. E-mail: [yonghui.sci@gmail.com](mailto:yonghui.sci@gmail.com).

**Methods and Note**

**Sampling, cultivation, and DNA sequencing**

The sampling site was the “Lille Firn” (LF) glacier (81.566° N, 16.363° W) in the Knuths Fjeld area of northeast Greenland, 5.6 km away from the Villum Research Station (VRS). The LF glacier was independently formed at the lee side of a small hill, surrounded by ~160 km^2^ land of permafrost. Surface ice was collected on 2 July 2018 using a sharp spear after removal of the top ~2 m thick snow cover. The sampled ice was processed within 24 hrs in the nearby VRS laboratory. The ice surface was cleaned with running pre-sterilized and cooled water before melting at 4 °C in sterile whirl-pak sampling bags (Nasco) for 24-48 hours. During our fieldwork, the melt season just began and the majority of the whole area was heavily covered by snow. There was a small patch of exposed soil (designated as ES), ca. 50 meters away to the north of the glacial sampling site, where a few kilograms of surface soil (top layer, a few centimeters thick) were collected into a sterile whirl-pak bag, kept at 4 °C as a comparison sample for the following cultivation and metagenomics analyses.

For bacterial cultivation, 100 μL 3.0 μm-prefiltered meltwater was plated onto 1/5 strength R2A agar (Difco) at the VRS laboratory. The plates were kept aerobically for 8 weeks under room temperature and then screened for bacteriochlorophyll fluorescence using a colony infrared imaging system as described in Zeng et al. (2014). A MALDI-TOF mass spectrometer (Microflex LT, Bruker Daltonics) was used to generate protein fingerprints for individual colonies as described previously (Zervas et al., 2019) in order to rapidly determine their relatedness at the species level. Genomic DNA of the selected isolates was extracted from cells harvested from 1/5 R2A agar plates after 2-week growth using the EasyPure bacterial genomic DNA kit (Transgen Biotech, Beijing, China) and was sequenced using both the BGISEQ sequencer (BGI Europe, Denmark) and an in house Nanopore MinION device following standard protocols as described previously (Zervas et al., 2019). Gap-free complete genomes were assembled using Unicycler (ver. 0.4.8) in a hybrid mode with default settings (Wick et al., 2017). Genomes were annotated with NCBI’s prokaryotic genome annotation pipeline. Genome synteny was visualized with the Easyfig program (ver. 2.2.3; Sullivan et al., 2011).

For amplicon and metagenomics analyses, cells in ~20 L of 3.0 μm-prefiltered melt ice were collected onto 0.2 μm membrane filters (Whatman) and the total DNA was extracted using the DNeasy PowerWater DNA extraction kit (Qiagen, Germany). Amplicon sequencing of 16S rRNA genes was conducted at BGI Hong Kong by targeting the V3-V4 region and the data were analyzed using the 16S pipeline embedded in the Geneious Prime (Biomatters, New Zealand). The generated PE reads were end-trimmed with the quality score set as Q>20 and then merged. Non-merged reads were discarded and only the high-quality merged reads were used for the community structure analysis. Total environmental DNA was sequenced on an Illumina NovaSeq platform at BGI Hong Kong. The generated ~220 G bases of PE reads (150 bp long) were end-trimmed (>Q20, >50 bases long) and assembled using Megahit (ver. 1.1.x; Li et al., 2015) with a minimum contig length of 500 bp. There were 948,300 contigs (⩾1 kb; total length, 2.69 G bases) assembled for the LF glacial sample and 2,834,721 contigs (⩾1 kb; total length, 5.63 G bases) for the ES soil sample.

**Binning, database mining, and bioinformatics**

The binning of metagenome-assembled contigs was performed using MetaBAT2 with default settings (Kang et al., 2019). Genomic bins were de-replicated using dRep (Olm et al., 2017) and quality-checked with CheckM by following the lineage-specific workflow (Parks et al., 2015). Only bins of good quality (>50% completeness, <10% contamination; recommended by Bowers et al., 2017) were included for further analysis. Each bin was taxonomically classified using the GTDB-Tk tool (https://github.com/Ecogenomics/GTDBTk, Parks et al., 2018). Based on the GTDB-Tk classification results, genomes that have more than 10% of markers with multiple hits were discarded.

The sequences of *pufM* genes (encoding the M protein of bacteriochlorophyll-containing type-2 reaction center), rhodopsin genes, and the single-copy gene *recA* were retrieved from the ES and LF metagenomic assemblies by tBLASTn search. The *recA* gene encodes a DNA recombination and repair protein and is commonly used as a phylogenetic biomarker, which is within a similar size range (234 AA) as above phototrophy-related genes (average length: *pufM*, 293 AA; rhodopsin gene, 201 AA). Multiple reference sequences of each gene with wide phylogenetic coverage were used as tBLASTn queries against the ES and LF metagenomic assemblies. The cutoff E-value, minimum identity, and coverage for tBLASTn hits were set as e^-5^, 30% sequence identity, and 30% query coverage, respectively. The lowest scored hit was confirmed as the target gene by the BLASTp search against NCBI’s RefSeq protein database. The cleaned tBLASTn results from different reference sequences were pooled for each gene and used for abundance estimates.

To assess the relative abundance of *pufM* and rhodopsin genes in the metagenomes, original reads were mapped onto the assembled contigs using Bowtie2 (Langmead and Salzberg, 2012) and SAMtools (Li et al., 2009). After duplicate reads were removed using the Picard toolkit (https://gatk.broadinstitute.org), mapped reads per target gene (*pufM*, rhodopsin, and *recA* genes with their locations on assemblies marked during tBLASTn analysis) were counted using *featureCounts* of the *Subread* package (Liao, et al., 2013) and were further normalized as the number of reads per million reads per kb length of the gene. The relative abundance of phototrophs in the whole community was estimated as the number of total reads mapped to a phototrophic gene divided by the number of total reads mapped to the single-copy *recA* gene.

Searching for rhodopsin and *pufM* genes in prokaryotic genomes deposited into public databases was carried out as follows. First, all retrievable prokaryotic genomes deposited into NCBI’s Microbial Genome database (n=215,874) and ENA’s WGS database (n=227,814) were bulk downloaded from NCBI and ENA’s FTP servers (as of 11 November 2019). Common human-associated bacteria (NCBI, n=107,120; ENA, n=129,669) were removed from the collection by searching for keywords in the *fasta* headers, including *Brucella*, *Chlamydia*, *Clostridioides*, *Clostridium*, *Corynebacterium*, *Enterococcus*, *Escherichia*, *Haemophilus*, *Helicobacter*, *Klebsiella*, *Listeria*, *Mycobacterium*, *Neisseria*, *Salmonella*, *Shigella*, *Staphylococcus*, *Streptococcus*, and *Yersinia*, and three highly represented species according to the stats in the GTDB genome collection (https://gtdb.ecogenomic.org/stats), i.e. *Acinetobacter baumannii*, *Pseudomonas aeruginosa*, and *Mycobacteroides abscessus*. Then, the ENA and NCBI genome datasets were merged (non-redundant, n=108,754) and served as the local BLAST database built with NCBI’s BLAST+ tools (ncbi-blast-2.2.18). The *pufM* and XR genes of the *Tardiphaga* isolates in this study (designated as Tar_pufM and Tar_XR) and the PR gene of *Pelagibacter* sp. IMCC9063 (Pel_PR) were used as tBLASTn queries. The initial thresholds of 30% sequence identity, 30% query coverage, and an E-value cutoff of e^-5^ were applied to filter the tBLASTn results. The tBLASTn hit with the lowest score was further checked by BLAST at the UniProt website until the lowest one was confirmed as the target gene.

**Rhodopsin gene classification**

To reduce the uncertainty when placing short sequences on phylogenetic trees, short sequenes were excluded from phylogenetic analysis but kept in read mapping and abundance estimates. The length cutoff was 150 AA for rhodopsin genes. The rhodopsin gene sequences together with references were aligned with MUSCLE (Edgar, 2004) and the phylogenetic tree was inferred by following Bulzu et al. (2019). Briefly, the identified rhodopsin sequences (>150 AA, n=775) were scanned with HMMER against a locally installed Pfam database (version 32) using the script pfam_scan.pl (obtained from the Pfam’s FTP site). As the rhodopsins proved to be composed of both type-1 (n=657 sequences) and heliorhodopsin (n=128 sequences), we merged them with a previously published database (n=410 sequences) (Bulzu et al., 2019). The rhodopsin sequences (n=1,185) were screened with PREQUAL (Whelan et al., 2018) in order to mask non-homologous characters, and aligned with the PASTA software (Mirarab et al., 2014) using default settings. A maximum-likelihood phylogeny was constructed using IQ-TREE (Nguyen et al., 2015) with the LG+F+G4 substitution model (chosen as the best-fitting model by ModelFinder) and 1000 ultrafast bootstrap replicates. The phylogenetic tree was used for the classification of rhodopsin genes.

**XR operon phylogeny**

The translated protein sequences of the six genes in the XR operon (XR-*crtEIBY*-*brp*) were concatenated and the generated protein sequence (2,060 amino acid sites) was used as the tBLASTn query against NCBI’s RefSeq genome database to search for closely related relatives. All hits that meet the threshold (total query coverage >80%, total sequence identity >50%, and individual gene’s sequence identity >30% and query coverage >50%) were downloaded from NCBI. The protein sequence of each gene in *Tardiphaga*’s XR operon was individually aligned with reference sequences using MUSCLE (Edgar, 2004). All alignments of the six genes were concatenated for phylogeny inference using FastTree (ver.2.1.12, LG model and gamma approximation with 100 bootstrap replicates; Price et al., 2010) within the Geneious Prime environment.

**Note on the negative results from the cultivation of *Tardiphaga* stains in liquid media and colony pigment analysis**

Growth of *Tardiphaga* strains in the liquid media was tested on either full-strength R2B (the liquid medium version of R2A) or 1/5 R2B for heterotrophic growth and on the Rhodospirillaceae medium (DSMZ medium 27 without using L-cysteiniumchloride and resazurin) that was designed for purple bacterial photoautotrophic growth. The cultivation conditions were 25°C, 16/8 hr light cycle with a 100 W tungsten lamp. For aerobic growth, cotton-plugged flasks were used with constant shaking at 200 rpm. For anaerobic growth, 125 mL glass serum bottles were used and the test medium was flushed with nitrogen gas and sealed with a rubber septum under a stream of nitrogen gas prior to autoclave at 121°C for 15 min. Sterile syringes were used to inoculate and remove samples and to inject temperature-sensitive components of the medium. No growth was observed as measured by changes in OD_600_ during an 11-week aerobic incubation and almost half a year of anaerobic growth. All four strains were tested and showed negative results.

For pigment analysis, colonies grown for three weeks on 1/5 R2A plates were scraped and the pigment was extracted with 100% methanol. Twenty microliters of the mix were injected into the Nexera LC-40 HPLC system (Shimadzu, Japan; accessed at Michal Koblížek’s group in the Institute of Microbiology CAS, Třeboň, Czech Republic) equipped with Kinetex 2.6 µm C8 100Å column (150 mm × 4.6 mm, Phenomenex) heated at 40°C. A binary solvent system was used: A, 25% 28 mM ammonium acetate + 75% methanol; B, 100% methanol at a constant flow rate of 0.8 mL min^-1^. BChl *a* peaks and carotenoids were observed at 770 nm and 490 nm respectively. To detect the pigment of salinixanthin that was involved in the light-harvesting carotenoid antenna of xanthorhodopsin (Balashov et al., 2005), *Salinibacter ruber* strain DSM 13855 was purchased from DSMZ and used as the positive control. No BChl and salinixanthin signals were observed.

**References**

Balashov SP, Imasheva ES, Boichenko, VA, Antón J, Wang JM, Lanyi JK (2005) Xanthorhodopsin: a proton pump with a light-harvesting carotenoid antenna. Science 309: 2061-2064.

Bowers RM, Kyrpides NC, Stepanauskas R, Harmon-Smith M, Doud D, Reddy TB, Schulz F, Jarett J, Rivers AR, Eloe-Fadrosh EA, Tringe SG (2017) Minimum information about a single amplified genome (MISAG) and a metagenome-assembled genome (MIMAG) of bacteria and archaea. Nature biotechnology 35(8):725.

Bulzu, P. A., Andrei, A. Ş., Salcher, M. M., Mehrshad, M., Inoue, K., Kandori, H., ... & Banciu, H. L. (2019). Casting light on Asgardarchaeota metabolism in a sunlit microoxic niche. Nature microbiology, 4(7), 1129-1137.

Edgar, R. C. (2004). MUSCLE: a multiple sequence alignment method with reduced time and space complexity. BMC bioinformatics, 5(1), 113.

Kang D, Li F, Kirton ES, Thomas A, Egan RS, An H, Wang Z. (2019) MetaBAT 2: an adaptive binning algorithm for robust and efficient genome reconstruction from metagenome assemblies. PeerJ 7:e27522v1.

Langmead, B., & Salzberg, S. L. (2012). Fast gapped-read alignment with Bowtie 2. Nature methods, 9(4), 357.

Li, H., Handsaker, B., Wysoker, A., Fennell, T., Ruan, J., Homer, N., ... & Durbin, R. (2009). The sequence alignment/map format and SAMtools. Bioinformatics, 25(16), 2078-2079.

Liao, Y., Smyth, G. K., & Shi, W. (2013). The Subread aligner: fast, accurate and scalable read mapping by seed-and-vote. Nucleic acids research, 41(10), e108-e108.

Mirarab, S., Nguyen, N., Guo, S., Wang, L. S., Kim, J., & Warnow, T. (2015). PASTA: ultra-large multiple sequence alignment for nucleotide and amino-acid sequences. Journal of Computational Biology, 22(5), 377-386.

Nguyen, L. T., Schmidt, H. A., Von Haeseler, A., & Minh, B. Q. (2015). IQ-TREE: a fast and effective stochastic algorithm for estimating maximum-likelihood phylogenies. Molecular biology and evolution, 32(1), 268-274.

Olm MR, Brown CT, Brooks B, Banfield JF (2017) dRep: a tool for fast and accurate genomic comparisons that enables improved genome recovery from metagenomes through de-replication. The ISME journal, 11(12):2864.

Parks DH, Chuvochina M, Waite DW, Rinke C, Skarshewski A, Chaumeil PA, Hugenholtz P (2018) A standardized bacterial taxonomy based on genome phylogeny substantially revises the tree of life. Nature Biotechnology, 36:996-1004.

Parks DH, Imelfort M, Skennerton CT, Hugenholtz P, Tyson GW (2015) CheckM: assessing the quality of microbial genomes recovered from isolates, single cells, and metagenomes. Genome research, 25(7):1043-55.

Price, M. N., Dehal, P. S., & Arkin, A. P. (2010). FastTree 2–approximately maximum-likelihood trees for large alignments. PloS one, 5(3).

Sullivan, M. J., Petty, N. K., & Beatson, S. A. (2011). Easyfig: a genome comparison visualizer. Bioinformatics, 27(7), 1009-1010.

Wick RR, Judd LM, Gorrie CL, Holt KE (2017) Unicycler: resolving bacterial genome assemblies from short and long sequencing reads. PLoS Computational Biology, 13(6), e1005595.

Zervas, A., Zeng, Y., Madsen, A. M., & Hansen, L. H. (2019). Genomics of Aerobic Photoheterotrophs in Wheat Phyllosphere Reveals Divergent Evolutionary Patterns of Photosynthetic Genes in *Methylobacterium* spp. Genome biology and evolution, 11(10), 2895-2908.
