# Supplementary material for: Potential Rhodopsin- and Bacteriochlorophyll-Based Dual Phototrophy in a High Arctic Glacier
Source: mBio. 2020 Nov 24;11(6):e02641-20. doi: 10.1128/mBio.02641-20 (PMC7701988; doi:10.1128/mBio.02641-20)
Supplement: FIG S3 [file mBio.02641-20-sf003.pdf]

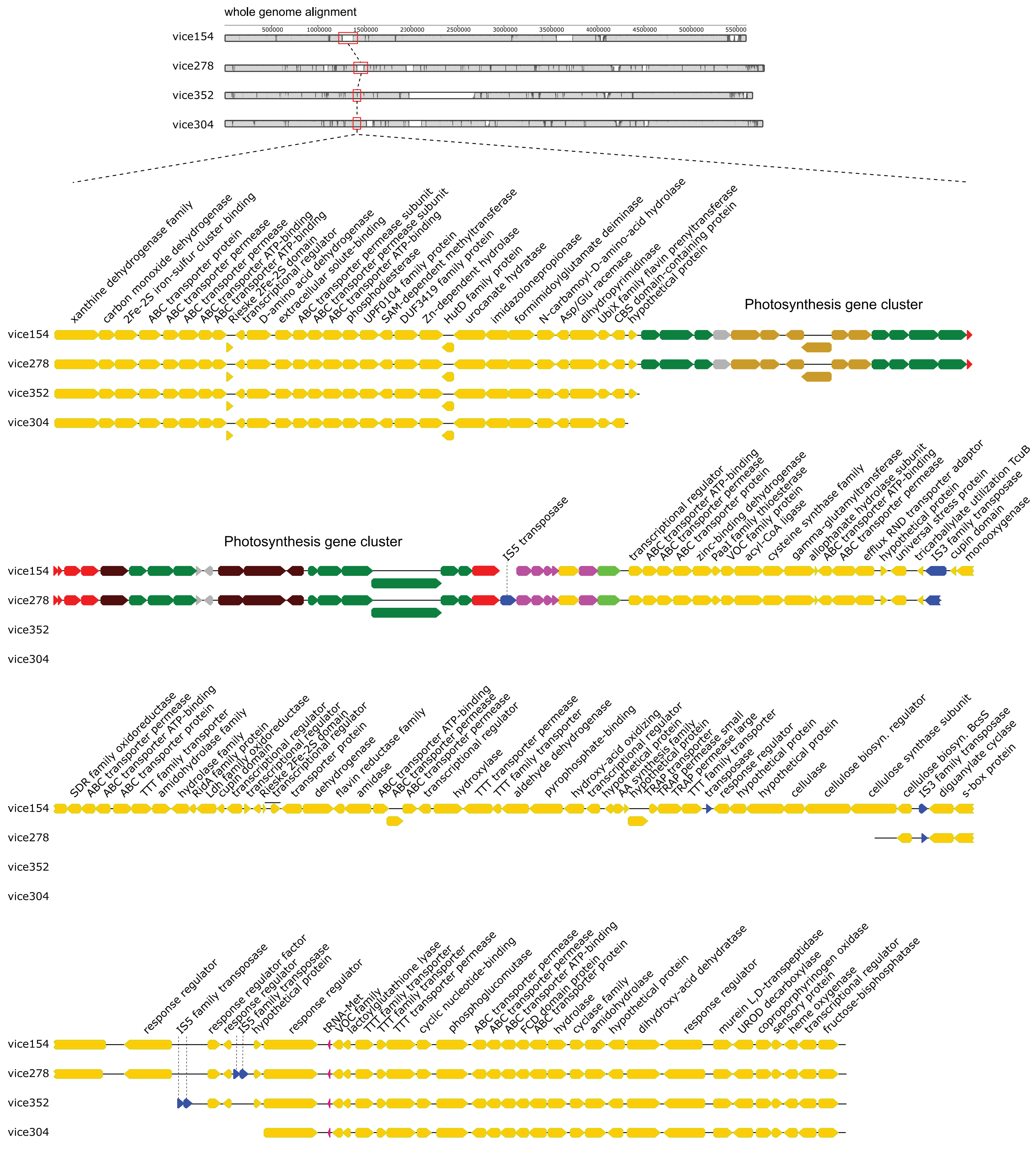


**Figure S3** Annotated genomic regions surrounding the photosynthesis gene cluster in *Tardiphaga* strains vice154 and vice278 in comparison to strains vice304 and vice352 that do not contain a photosynthesis gene cluster.
