# Supplementary material for: Potential Rhodopsin- and Bacteriochlorophyll-Based Dual Phototrophy in a High Arctic Glacier
Source: mBio. 2020 Nov 24;11(6):e02641-20. doi: 10.1128/mBio.02641-20 (PMC7701988; doi:10.1128/mBio.02641-20)
Supplement: TABLE S4 [file mBio.02641-20-st004.pdf]

Table S4 Survey of key functional genes with biogeochemical or energetic importance in the genomes of *Tardiphaga* isolates. The gene list was complied from the FunGen pipeline (http://fungene.cme.msu.edu/) and the authors' own collection.

Category	Gene	Enzyme	vice154	vice278	vice304	vice352
C metabolism	scd2	esterase / lipase	●	●	●	●
C metabolism	xylA	xylose isomerase	○	○	○	○
One carbon metabolism	cooS	carbon monoxide dehydrogenase	○	○	○	○
One carbon metabolism	pmoA	particulate methane monooxygenase A-subunit	○	○	○	○
One carbon metabolism	pxmA1	particulate methane monooxygenase beta subunit	○	○	○	○
One carbon metabolism	prk	phosphoribulokinase	●	●	●	●
One carbon metabolism	cbbL	ribulose-bisphosphate carboxylase large subunit	●	●	●	●
One carbon metabolism	cbbM	ribulose-bisphosphate carboxylase small subunit	●	●	●	●
One carbon metabolism	smmo	soluble methane monooxygenase	●	●	●	●
N metabolism	amiE	aliphatic amidase	●	●	●	●
N metabolism	amoA	ammonia monooxygenase subunit A	○	○	○	○
N metabolism	ansA	asparaginase	○	○	○	○
N metabolism	aspA	aspartate ammonia-lyase	○	○	○	○
N metabolism	glsA	glutaminase	●	●	●	●
N metabolism	hutH	histidine ammonia-lyase	○	○	○	○
N metabolism	norB	nitric oxide reductase	○	○	○	○
N metabolism	p450nor	nitric oxide reductase (NAD(P), nitrous oxide-forming)	○	○	○	○
N metabolism	nir	nitrite reductase	●	●	●	●
N metabolism	nifH	nitrogenase iron protein	○	○	○	○
N metabolism	anfD	nitrogenase iron-iron protein, alpha chain	○	○	○	○
N metabolism	nifD	nitrogenase molybdenum-iron protein subunit alpha	○	○	○	○
N metabolism	vnfD	nitrogenase vanadium-iron protein alpha chain	○	○	○	○
N metabolism	nosZ	nitrous oxide reductase catalytic subunit	○	○	○	○
N metabolism	napA	periplasmic nitrate reductase catalytic subunit	●	●	●	●
N metabolism	napE	periplasmic nitrate reductase subunit	●	●	●	●
N metabolism	apr	serine 3-dehydrogenase	○	○	○	○
N metabolism	ureC	urease	●	●	●	●
P metabolism	phy	4-phytase	●	●	●	●
P metabolism	alp	alkaline phosphatase	●	●	●	●
P metabolism	phnX	phosphonoacetaldehyde dehydrogenase	○	○	○	○
P metabolism	PPK	polyphosphate kinase	●	●	●	●
S metabolism	dsrA	dissimilatory sulfite reductase alpha subunit	●	●	●	●
S metabolism	soxB	sulfur oxidation protein / thiosulfohydrolase	●	●	●	●
Plant material utilization	cbh1	cellobiohydrolase	○	○	○	○
Plant material utilization	chiA	chitinase	○	○	○	○
Plant material utilization	chb	chitobiase	○	○	○	○
Plant material utilization	ligE	glutathione S-transferase	○	○	○	○
Plant material utilization	lcc	laccase	○	○	○	○
Plant material utilization	lip	lignin peroxidase	○	○	○	○
Plant material utilization	appA	phosphoanhydride phosphohydrolase	○	○	○	○
Protein catabolism	npr	bacillolysin	○	○	○	○
Protein catabolism	sub	subtilisin	○	○	○	○
Protein catabolism	trp	trypsin	●	●	●	●
Phototrophy	acsF	magnesium-protoporphyrin IX monomethyl ester aerobic oxidative cyclase	●	●	●	●
Phototrophy	pufL	photosynthetic reaction center subunit L	●	●	●	●
Phototrophy	pufM	photosynthetic reaction center subunit M	●	●	●	●
Phototrophy	rho	rhodopsin	●	●	●	●
Oxygen fluctuation	ctaD	cytochrome c oxidase subunit, aa3-type, low affinity	●	●	●	●
Oxygen fluctuation	cydA	cytochrome bd-I ubiquinol oxidase subunit 1, bd-type, high affinity	●	●	●	●
Oxygen fluctuation	cyoB	cytochrome bo(3) ubiquinol oxidase subunit, bo3-type, low affinity	●	●	●	●
Oxygen fluctuation	cbaA	cytochrome c oxidase subunit, ba3-type, high affinity	●	●	●	●
Oxygen fluctuation	fixN	cytochrome c oxidase subunit, cbb3-type, high affinity	●	●	●	●
Oxygen fluctuation	ccoN	cytochrome c oxidase subunit, cbb3-type, high affinity	○	○	○	○
Sense and regulation	PAP	serine/threonine protein phosphatase	●	○	●	●
Sense and regulation	PTP	tyrosine protein phosphatase	●	●	●	●
Other - acetate metabolism	acs	acetyl-CoA synthetase	○	○	○	○
Other - animal material utilization	col	collagenase	○	○	○	○
Other - arginine catabolism	rocF	arginase	●	●	●	●
Other - folate metabolism	cpg	glutamate carboxypeptidase	○	○	○	○
Other - glycan metabolism	nag3	beta-hexosaminidase	○	○	○	○
Other - H metabolism	hydA	iron hydrogenase	○	○	○	○
Other - H_2O_2 generation	glx	glyoxaloxidase 1	○	○	○	○
Other - methanogenesis	mcrA	methyl-coenzyme M reductase, alpha subunit	○	○	○	○
Other - oligosaccharides metabolism	exc1	beta-N-acetylglucosaminidase	○	○	○	○
Other - phenol degradation	ppo	phenoloxidase	○	○	○	○
Other - purine metabolism	add	adenosine deaminase	○	○	○	○
